# Acute Gastric Volvulus in the Setting of a Paraesophageal Hernia With Hemoperitoneum: Emergency Department Diagnosis and Management

**DOI:** 10.7759/cureus.20404

**Published:** 2021-12-14

**Authors:** Derrick Huang, Michael Hughes, Samyr Elbadri, Michael Falgiani, Latha Ganti

**Affiliations:** 1 Emergency Medicine, Ocala Regional Medical Center, Ocala, USA; 2 Emergency Medicine, Envision Physician Services, Plantation, USA; 3 Emergency Medicine, University of Central Florida College of Medicine, Orlando, USA; 4 Emergency Medicine, HCA Healthcare Graduate Medical Education, Olrando, USA

**Keywords:** abdominal pain, hemoperitoneum, paraesophageal hernia, emergency medicine, gastric volvulus

## Abstract

Acute gastric volvulus is an uncommon emergency department (ED) presentation associated with high mortality from gastric ischemia and perforation. The diagnosis of this pathology is complicated by its intermittent symptoms and similarity in presentation to more common disorders encountered in the ED. Assessing for key risk factors, such as the presence of a hiatal hernia, and the use of expeditious imaging modalities, such as bedside radiography and point-of-care ultrasonography, are essential in rapid diagnosis and time-sensitive, definitive surgical intervention.

## Introduction

Gastric volvulus is a rare, potentially life-threatening pathology that occurs when the stomach is abnormally rotated along its long or short axis [1]. This rotation causes varying degrees of gastric outlet obstruction and may present intermittently, which can result in chronic abdominal symptoms, such as dyspepsia, bloating, vomiting, dysphagia, and chronic anemia from upper gastrointestinal bleeding [2,3]. When the rotation is greater than 180°, gastric volvuli can present acutely with complete gastric outlet obstruction, classically resulting in Borchardt's triad of vomiting, epigastric pain, and an inability to pass a nasogastric tube (NGT) - a combination found in 70% of cases [1]. This severe vomiting may be nonproductive and associated with hematemesis as well as a peritoneal abdomen from mucosal ischemia [1,3]. Complete gastric outlet obstruction may carry a mortality rate as high as 30% due to gastric ischemia or perforation [4].

In the emergency department (ED), the diagnosis of gastric volvulus is difficult due to its rarity and similar symptomatology to more common ED presentations in both the acute and chronic forms of the disease. For example, acute presentations may be confused with pancreatitis and acute coronary syndrome, whereas intermittent, chronic presentations may be falsely attributed to peptic ulcer disease, gallbladder pathologies, and gastroesophageal reflux disease [3,5]. Here, we present a rare case of an acute gastric volvulus in the setting of a paraesophageal hernia with hemoperitoneum requiring emergent exploratory laparotomy.

## Case presentation

A 75-year-old woman with a past medical history of hypertension, hyperlipidemia, stroke without residual deficits, appendectomy, laparoscopic repair of a hiatal hernia two years prior, and an anaphylactic allergy to contrast dye presented to the ED with left-sided abdominal pain that started one day prior to arrival. She reported that the pain started on the left side of her left abdomen and then progressed to include the right side of her abdomen. Her pain was associated with retching and vomiting that was non-bilious, non-bloody, and progressed to dry heaving. The patient reported her last bowel movement occurred on the morning prior to arrival and was normal. Notably, she had a milder episode of similar symptoms about 6 months ago; however, she elected not to present to the hospital because the pain subsided. She denied a history of recent trauma, coronary artery disease, and use of any blood thinner mediations.

On her initial vitals, the patient had pulse oximetry of 98% on room air, blood pressure of 106/58 mmHg, temperature of 98°F, respiratory rate of 18, and heart rate of 86. The patient was in no apparent distress. She had a kyphotic habitus, a regular rate and rhythm, clear lung sounds, and normal pulses in all extremities. On her abdominal exam, she was non-distended with upper abdominal rigidity and severe tenderness to palpation. Laboratory evaluation revealed a white blood cell count of 7.0 x10^9^/L, hemoglobin/hematocrit of 10.1/31.7, and platelet count of 226 x10^9^/L. Her lactic acid and lipase levels were 2.0 mmol/L and 85 U/L, respectively. The electrocardiogram, troponin, electrolyte, and hepatic tests were unremarkable.

**Figure 1:**
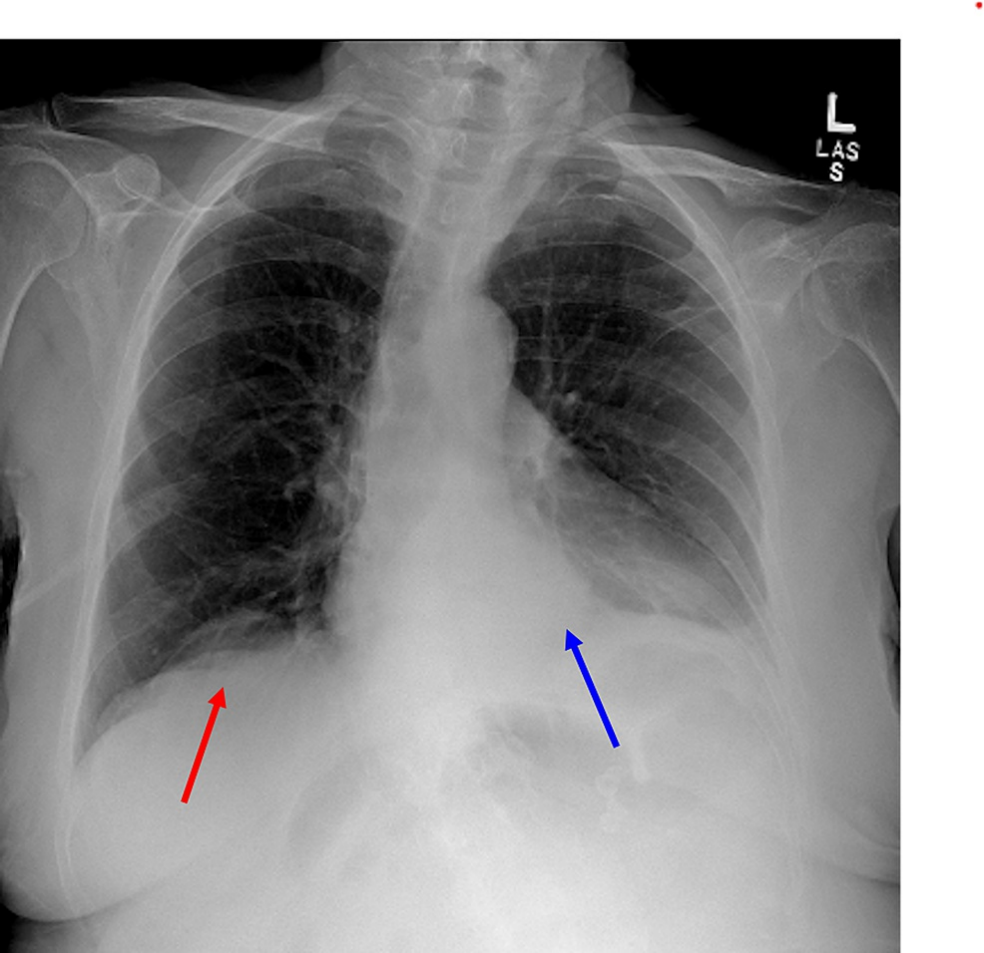
Chest radiography remarkable for a moderate size hiatal hernia (blue arrow), eventration of the right hemidiaphragm (red arrow), left basilar density with blunting of the left costophrenic angle, and scoliosis

On initial imaging, bedside chest radiography was remarkable for a moderate size hiatal hernia, left basilar density with blunting of the left costophrenic angle, eventration of the right hemidiaphragm, and scoliosis (Figure 1).

**Figure 2:**
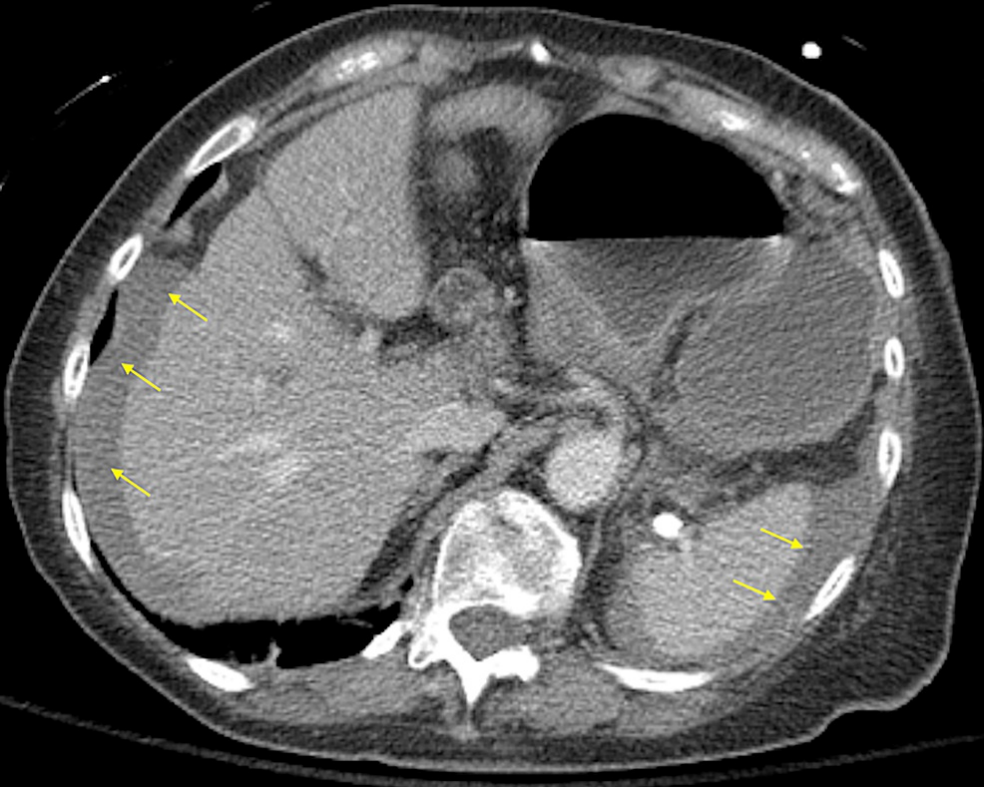
CT with contrast angiography with an axial view of a large hiatal hernia with suspected organo-axial volvulus and free fluid surrounding the distal esophagus with moderate hemoperitoneum surrounding the stomach, liver, and spleen (yellow arrows)

**Figure 3:**
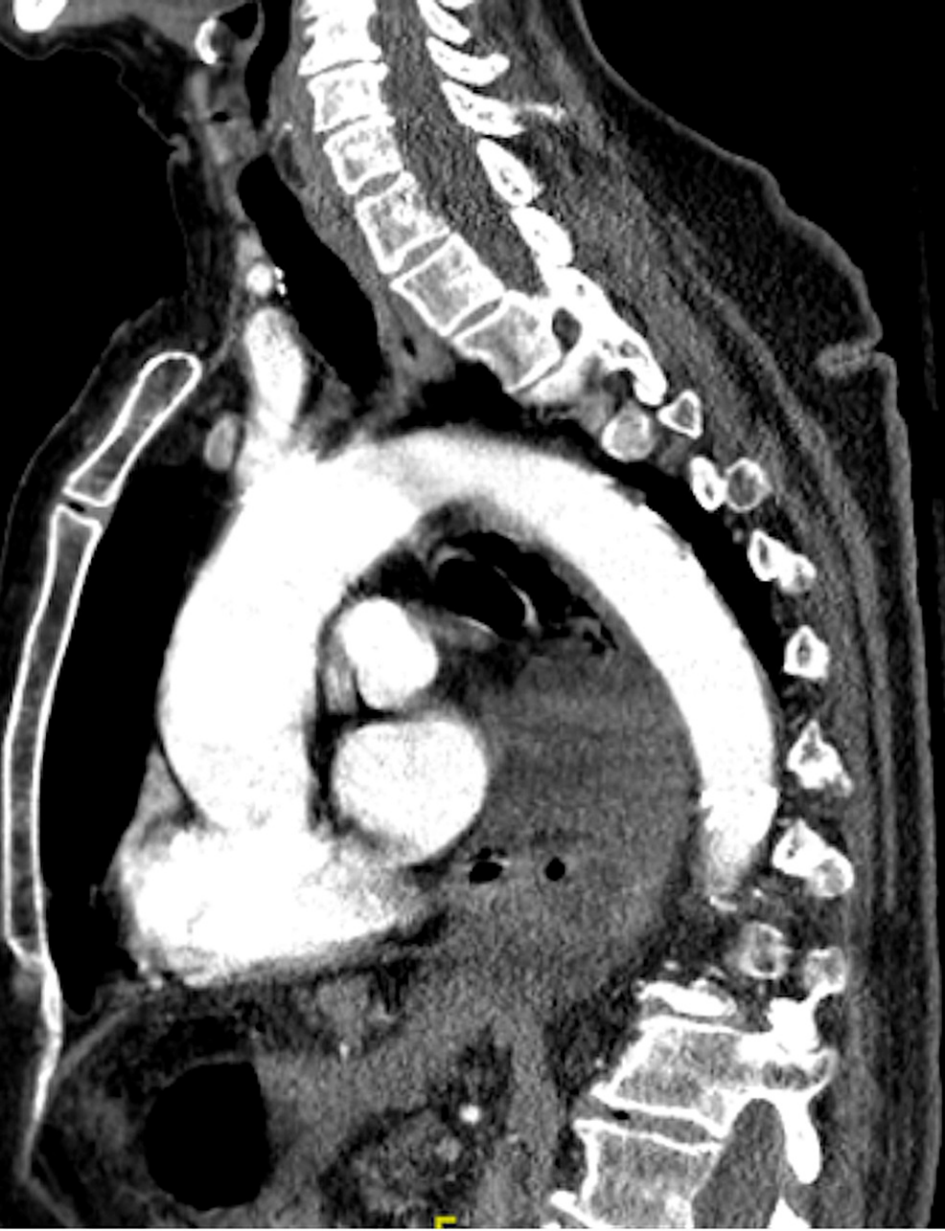
CT with contrast angiography with a sagittal view of a large hiatal hernia with suspected organo-axial volvulus and free fluid surrounding the distal esophagus

**Figure 4:**
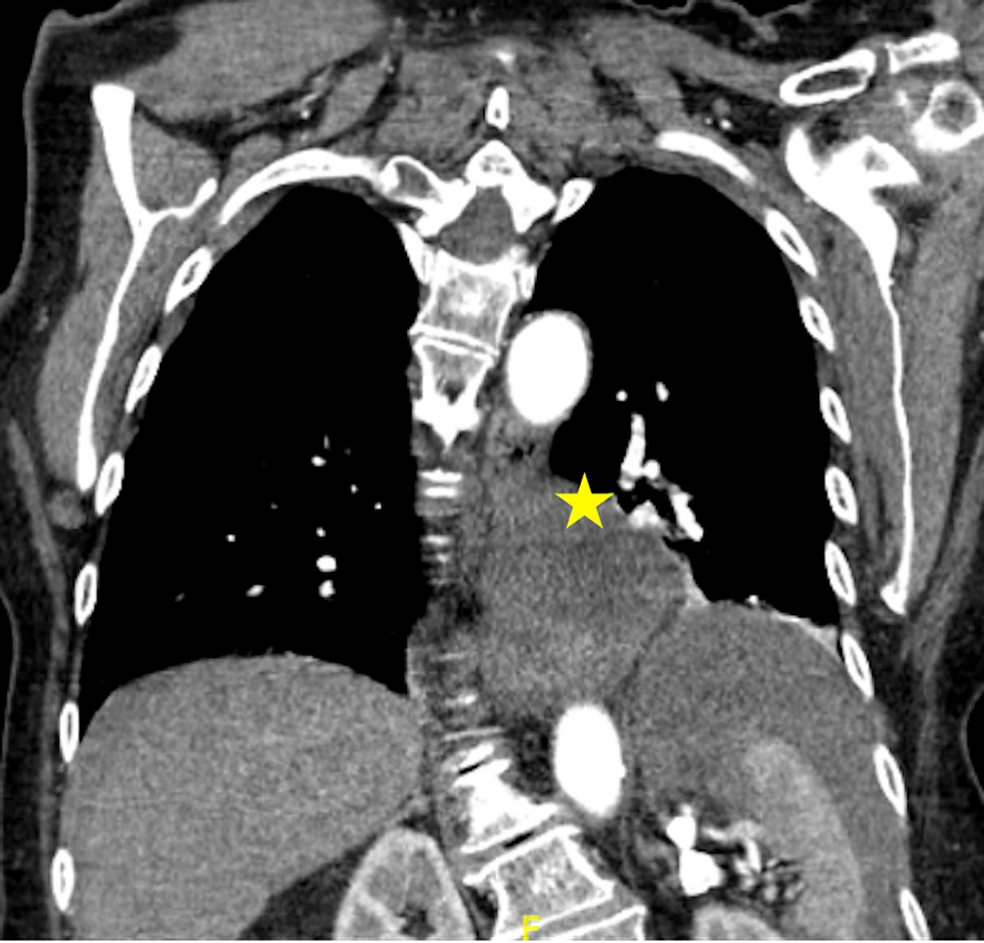
CT with contrast angiography with a coronal view of a large hiatal hernia (star) with suspected organo-axial volvulus and free fluid surrounding the distal esophagus with moderate hemoperitoneum surrounding the liver and spleen

Point-of-care ultrasound (POCUS) was remarkable for free fluid most prominent in the suprapubic window. The surgical team was immediately consulted and the patient was administered broad-spectrum antibiotics and intravenous (IV) fluid resuscitation. Given the patient’s reported history of anaphylactic allergy to contrast dye, the patient was pre-medicated with IV methylprednisolone and diphenhydramine prior to computed tomography (CT) imaging. CT with contrast angiography was remarkable for a large hiatal hernia with suspected organo-axial volvulus and free fluid surrounding the distal esophagus with moderate hemoperitoneum surrounding the stomach, liver, spleen, and deep pelvis without evidence of active arterial extravasation (Figures 2-4).

The patient was immediately taken to the operating room for NGT placement and exploratory laparotomy. The patient was found to have about 2 L of hemoperitoneum and a paraesophageal hernia recurrence, which was reduced and repaired with mesh, and a gastric volvulus, which was repaired with gastropexy. The patient was transferred to the intensive care unit for post-operative care and was ultimately downgraded with a good prognosis for recovery.

## Discussion

We report a case of an acute gastric volvulus in the setting of a paraesophageal hernia with hemoperitoneum. Our case was complicated by its likely early presentation and rapid progression of acute on chronic gastric volvulus as underscored by the patient’s recent bowel movement and unremarkable initial laboratory values. Importantly, our patient possessed multiple key risk factors for a gastric volvulus, including an age >50, a history of a paraesophageal hernia, diaphragmatic eventration on chest radiography (congenital thinning of the diaphragmatic musculature), and kyphoscoliotic anatomy. General risk factors include a history of diaphragmatic anomalies and any hiatal hernia (encompassing both sliding and paraesophageal hernias), phrenic nerve paralysis, and other anatomic stomach or splenic abnormalities [6]. The patient’s risk factors, peritoneal upper abdomen, and POCUS findings of free fluid were critical in both pursuing the CTA, which proved definitive in diagnosis, and intervening through an emergent, open laparotomy over laparoscopic or more conservative approaches to treatment. 

**Figure 5:**
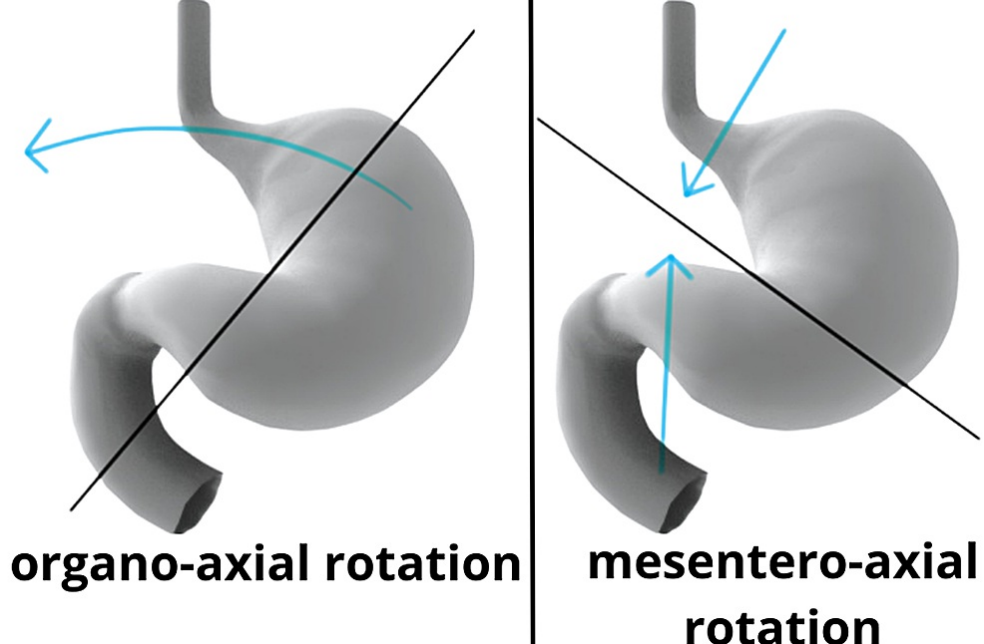
Types of gastric volvuli Adapted from: https://radiopaedia.org/cases/19257

Gastric volvuli have multiple modes of rotation that are associated with different presentations (Figure 5).

In our case, the patient’s volvulus was organo-axial in rotation, whereby the stomach rotates around its long axis along the gastroesophageal junction and the pylorus, where the antrum rotates anterosuperiorly and the fundus rotates posteroinferiorly resulting in an inverted position [6]. The organo-axial rotation occurs in about 60% of gastric volvulus cases and is more often associated with secondary etiologies, such as paraesophageal hernias, diaphragmatic hernias, and diaphragmatic eventration as well as a greater chance of stomach strangulation [6-9]. With mesentero-axial volvulus, the stomach rotates around its short axis with the antrum displaced above the gastroesophageal junction. This type of rotation is usually partial (<180°) and its etiology is typically primary in nature [6].

Multiple imaging modalities may be employed in the diagnosis of gastric volvulus. The gold standard test is a barium swallow, which has very high sensitivity and specificity for diagnosing a gastric volvulus; however, this modality may not be feasible in the ED setting [1]. In contrast, although it has more limited sensitivity and specificity, chest radiography is expeditious and may function as an effective initial screen. In our case, chest radiography was instrumental in identifying key risk factors of a hiatal hernia, seen as a retrocardiac opacity, as well as diaphragmatic eventration [10]. In addition, with a sensitivity and specificity of 64%-98% and 86%-100%, for the detection of free intraperitoneal fluid, abdominal US is a useful adjunct in assessing for complications such as hemoperitoneum [11]. Ultrasonography can also confirm the presence of a superficial thoracic hernia [10]. CT imaging in the setting of a suspected volvulus is slower to employ and will require contrast, but can prove definitive in diagnosis, assessment of complications, and providing key information to facilitate successful surgical intervention [10]. CT with angiography may be required if there is a concern for hemoperitoneum as seen in our case, in which free fluid was found on POCUS.

In the ED, the initial approach to patients with upper abdominal pain and vomiting includes stabilization and simultaneous assessment of a wide array of life-threatening etiologies. In particular, acute coronary syndrome, aortic dissection, and abdominal pathologies including acute mesenteric ischemia, aortic rupture, gastrointestinal perforation, and bowel obstruction can each present similarly and involve different intervention pathways. Given the wide differential diagnosis, the use of rapid imaging modalities such as portable chest radiography and POCUS is essential in narrowing the differential and rapid mobilization of surgical resources in preparation for definitive care. Once the diagnosis of gastric volvulus is made, placement of an NGT is an option in cooperative patients for gastric decompression in order to reduce stomach tension, which may improve perfusion and decrease the risk for gastric ischemia and necrosis [12]. In addition to IVF resuscitation, empiric broad-spectrum antibiotics are administered due to a concern for gastric perforation. Proton pump inhibitors may also be administered to reduce the volume of gastric secretions [13]. Ultimately, definitive treatment of acute gastric volvulus usually requires detorsion with laparoscopic surgery or open laparotomy and prevention with anterior gastropexy [1].

## Conclusions

Acute gastric volvulus is associated with high mortality from gastric ischemia and perforation. In the ED, narrowing down the wide differential diagnosis associated with the nonspecific presentation of gastric volvulus is dependent on an astute assessment of risk factors and clinical examination as well as rapid imaging modalities, such as bedside radiography and POCUS. Efficient diagnosis and immediate surgical consultation are essential in facilitating time-sensitive, definitive surgical intervention.
